# Antiviral Hammerhead Ribozymes Are Effective for Developing Transgenic Suppression of Chikungunya Virus in *Aedes aegypti* Mosquitoes

**DOI:** 10.3390/v8060163

**Published:** 2016-06-09

**Authors:** Priya Mishra, Colleen Furey, Velmurugan Balaraman, Malcolm J. Fraser

**Affiliations:** Department of Biological Sciences, Eck Institute for Global Health, University of Notre Dame, P.O. Box 369, Notre Dame, IN 46556, USA; Priya.Mishra.8@nd.edu (P.M.); cfurey1@nd.edu (C.F.); balaramanv@missouri.edu (V.B.)

**Keywords:** chikungunya, hammerhead ribozymes, transgenic mosquitoes, genetic control, antiviral strategy

## Abstract

The chikungunya virus (CHIKV) is an emerging pathogen with widespread distribution in regions of Africa, India, and Asia that threatens to spread into temperate climates with the introduction of its major vector, *Aedes albopictus*. CHIKV causes a disease frequently misdiagnosed as dengue fever, with potentially life-threatening symptoms that can result in a longer-term debilitating arthritis. The increasing risk of spread from endemic regions via human travel and commerce and the current absence of a vaccine put a significant proportion of the world population at risk for this disease. In this study we designed and tested hammerhead ribozymes (hRzs) targeting CHIKV structural protein genes of the RNA genome as potential antivirals both at the cellular and *in vivo* level. We employed the CHIKV strain 181/25, which exhibits similar infectivity rates in both Vero cell cultures and mosquitoes. Virus suppression assay performed on transformed Vero cell clones of all seven hRzs demonstrated that all are effective at inhibiting CHIKV in Vero cells, with hRz #9 and #14 being the most effective. *piggyBac* transformation vectors were constructed using the *Ae. aegypti* t-RNA^val^ Pol III promoted hRz #9 and #14 effector genes to establish a total of nine unique transgenic Higgs White Eye (HWE) *Ae. aegypti* lines. Following confirmation of transgene expression by real-time polymerase chain reaction (RT-PCR), comparative TCID_50_-IFA analysis, *in situ* Immuno-fluorescent Assays (IFA) and analysis of salivary CHIKV titers demonstrated effective suppression of virus replication at 7 dpi in heterozygous females of each of these transgenic lines compared with control HWE mosquitoes. This report provides a proof that appropriately engineered hRzs are powerful antiviral effector genes suitable for population replacement strategies

## 1. Background

Chikungunya virus (CHIKV) is a positive-sense RNA virus (Family *Togaviridae*) first described during an outbreak in southern Tanzania in 1952, and has been identified in almost 40 countries to date. It is currently endemic to Asia, India, and parts of Africa, and is propagated by the bite of female *Aedes* mosquitoes, principally *Ae. aegypti* and *Ae. albopictus*. The virus produces a disease that is characterized by abrupt onset of fever, muscle pain, nausea, headache, fatigue, rashes and debilitating joint pain [1]. Other complications associated with CHIKV include myocarditis, hepatitis, and ocular and neurological disorders [2]. 

Recent epidemics include La Reunion Island in 2005–2006, with 255,000 cases among a total population of 750,000 [3], India in 2006–2007, with 1.4 to 6.5 million estimated cases [4], and the Philippines in 2013, with 180 cases. Cases of CHIKV have also been reported in parts of Europe as a result of infected individuals traveling from endemic regions [5], and most recently in Caribbean island countries, and North and South America [6,7]. 

A significant amount of work is underway to develop a vaccine for CHIKV. This includes strategies such as chimeric vaccines [8], recombinant CHIKV vaccines [9], adenovirus-based vaccines [10], virus-like particles [11], and live attenuated vaccines [12]. However, even with a viable vaccine, there remain significant hurdles to effective prevention and control of any disease, especially in underdeveloped countries.

Alternative strategies are being explored to combat CHIKV through transgenic modification of the vector mosquitoes to produce refractoriness for virus transmission. Antiviral strategies including RNA interference (RNAi) employing small interfering RNAs (siRNA) [13] and small hairpin RNAs (shRNA) [14] have received the most attention. Although RNAi is an effective mechanism to suppress viral replication in mosquitoes [15,16], this strategy does have some limitations. RNAi must target highly conserved sequences of at least 21–23 nucleotides, which, in the case of CHIKV, limits the potential number of suitable target sites available. A single point mutation in this target site can result in escape mutants [17], and the longer the target site, the more potential there is for these escape mutations. RNAi requires the continual synthesis of a large amount of dsRNA in order to activate and maintain the RNAi machinery and effectively suppress viral replication [18], and some viruses may replicate at a rate that overcomes the RNAi response [19]. A recent report suggests that RNAi becomes unstable at lower temperatures, making temperature variations in natural mosquito habitats an important factor in the effective application of this approach [20].

Many of these limitations may be overcome using hRzs. These ribozymes are capable of targeting smaller sequences (15–16 nt), increasing the number of possible target sites and decreasing the probability of escape mutations. These hRzs are also capable of some degree of turnover, and do not require existing host cell machinery to be active. Finally, temperature stability is not a significant factor limiting the activity of these hRzs [21]. hRzs have been successfully employed to inhibit replication of hepatitis B virus [22], hepatitis C virus [23], human immunodeficiency virus (HIV) [24], and dengue virus [25].

In this report we designed seven hRzs based upon genome alignment analyses of 100 different CHIKV strains obtained from the GeneBank database. All of these ribozymes target the structural protein region of the virus. We constructed hygromycin selectable transformation vectors expressing each of our seven hRzs by fusion with an *Ae. aegypti* t-RNA^val^ Pol III promoter upstream of the hRz, and a downstream poly A_(60)_ tail. Each ribozyme was examined for its effectiveness in suppressing the vaccine strain of CHIKV (181/25) in sorted transformed clonal cultures. Analysis of CHIKV suppression using TCID_50_-IFA, quantitative PCR (qRT-PCR), and caspase 3 assays demonstrated that all seven of the hRzs exhibited complete suppression of CHIKV in sorted transformed cell clones, with hRz#9 and #14 being the most effective at a higher multiplicity of infection (MOI). 

We generated several transgenic *Ae. aegypti* mosquito lines expressing hRz #9 and #14 using the *piggyBac* transposon vector to examine their potential as antivirals for population replacement strategies. Our results demonstrate that each of the transgenic lines significantly reduced replication and salivary gland titers of CHIKV in heterozygous females, with six lines exhibiting no detectable virus in any individuals at 7 dpi compared to control HWE. The results confirm the utility of these hRzs as effective antiviral transgenes for potential use in eliminating the spread of CHIKV.

## 2. Materials and Method

### 2.1. Selection of hRz Target Sites in the CHIKV Genome

hRzs were designed based on CLUSTALX alignments of 100 CHIKV strains available from the GeneBank database. Several conserved 16–17 nucleotide sequences having a central GUC triplet cleavage site and flanking arms of 8–9 bp were selected. The sequences of each of the hRzs are present in Table 1.

### 2.2. Plasmid Construction

The hRz expression plasmid (pLAeRz#ARH, 25) was derived from the pQCXIH retroviral vector (Clontech, Mountain View, CA, USA) by cloning a Rous sarcoma virus (RSV)-driven hygromycin expression cassette [25] for selection. Each hRz encoding sequence, flanked by the *Ae. aegypti* t-RNA^val^ Pol III promoter and a downstream poly A_(60)_ tail, was positioned within the multiple cloning site of this plasmid (Supplementary Figure S1A). 

Template sequences for each hRz were synthesized (Invitrogen, Waltham, MA, USA) containing a portion of the t-RNA^val^ Pol III promoter sequence, the entire hRz sequence, and a portion of the poly A_(60)_ sequence (Table 1). These template sequences were then PCR amplified using a common set of primers, with the forward primer containing the entire t-RNA^val^ Pol III promoter sequence (ttattaaaggatccaccgttggtttccgtagtgtagtggttatcacgtctgcttcacacgcagaaggtccccggttcgacccgggcactacaaaaac) and the reverse primer containing the entire poly A_(60)_ tail (ggatctagagcggccgcaaaaaattttttttttttttttttttttttttttttttttttttttttttttttttttttttttttcctgcagg). With this common primer set the t-RNA^val^ Pol III promoter and poly A_(60)_ sequence were cloned upstream and downstream, respectively, of each hRz. These PCR amplified 210 bp fragments were gel purified, digested using BamHI and NotI enzymes, and cloned into the pLAeRz#ARH vector backbone. 

A cytomegalovirus (CMV)-promoted DsRed marker gene was PCR-amplified from the CMV DsRed express plasmid (Clontech, Mountain View, California, USA) using a forward primer containing the CMV promoter sequence (tttttttttgggccctagttattaatagtaatcaattacgg) and a reverse primer (tttttttttgggcccgcagtgaaaaaaatgctttatttgtc) complimentary to the DsRed marker gene.

The desired 1.5 kb band was gel-purified and cloned into the pLAeRz#ARH plasmid backbone downstream of the hRz + poly A_(60)_ tail using NotI and PspomI. 

The *piggyBac*-based, eye-specific fluorescence gene transduction plasmid pXL-BacII-3xP3-ECFP [26] was used to construct *Aedes* t-RNA^val^ Pol III promoted hRz #9 and #14 expression plasmids by PCR amplification from pLAeRz#9ARH and pLAeRz#14ARH plasmids, respectively, using a t-RNA^val^ forward primer, tttaaatttccgcggaccgttggtttccgtagtgtagtgg, and a reverse primer complementary to the pLAeRz#ARH plasmid sequence, tttaaatttagatcttgaggggatctgcggccg, containing SacII and BglII restriction sites, respectively (Supplementary Figure S1B). The plasmid used for amplification lacks the CMV-DsRed sequence. The 200 bp PCR products were purified from 2% gels and both the purified PCR products and the pXL-BacII-3xP3-ECFP vector were digested using SacII and BglII, column purified (Promega, Madison, WI, USA) and ligated to position hRz #9 and #14 within pXL-Bac-3xP3-ECFP downstream of the 3xP3-ECFP marker.

### 2.3. Cell Cultures, Transfection, Clonal Generation and Infection

Vero cells obtained from ATCC (Manassas, VA, USA) were maintained on complete DMEM (Sigma, St. Louis, MO, USA), which included 10% FBS (Atlanta Biological, Flowery Branch, GA, USA) and 1× non-essential amino acids (Gibco, Grand Island, NY, USA). Cells were plated at 1 × 10^5^/well and incubated overnight at 37 °C prior to transfection. The overnight cultures were washed twice with serum free media and then 1 µg of a given hRz expression plasmid mixed with lipofectimine plus and LTX reagent (Invitrogen, Grand Island, NY, USA) was applied. At 24 hours post transfection (hpt) the transfection mix was replaced by complete DMEM, and 48 hpt the cells were placed under hygromycin selection (200 µg/mL). Transformed cultures expressing each hRz were stabilized after three to four weeks on 200 µg/mL hygromycin (Invitrogen).

Cells were prepared for sorting by trypsinization following four weeks of selection with 200 µg/ml hygromycin, pelleted by centrifugation at 8000 rpm for 5 min, and washed by resuspension with gentle pipetting in 1x PBS, followed by centrifugation and resuspension in 1X PBS with 2% FBS + Penstrep + Gentamycin. Transformed and hygromycin selected cell populations were sorted as single cells into 96-well plates using FACSAria III (BD, Biosciences, San Jose, CA, USA). The sorted cells were allowed to attach for 24 h, followed by replacement of the media with a 1:1 mixture of complete DMEM and seven-day-old spent media. The sorted cells were then allowed to expand for one week.

The expanded cell clones were transferred to 12-well plates and allowed to grow for two weeks. Clones for each hRz were plated in six-well plates at a density of 2 × 10^5^/well for 24 h before infection. Clones were challenged with virus for 2 h, the media was replaced with complete DMEM, and the infections were allowed to progress for seven days at 37 °C. Each clone was screened for the characteristic cytopathic effect (CPE) compared to the untransformed wild-type Vero cells. The clones lacking visible CPE were further analyzed by TCID_50_-IFA, caspase 3 and qRT-PCR following challenge to confirm the absence of CHIKV.

### 2.4. RT-PCR Analysis

hRz-expressing cells lines were trizol (Invitrogen) extracted and the concentration of total cellular RNA was spectrometrically assayed. A total of 5 µg RNA was DNase treated for each sample and RT-PCR was performed using the Superscript III One-Step RT-PCR kit (Invitrogen). A common t-RNA^val^ forward primer and hRzs specific reverse primers (Supplementary Table S1) were utilized for both RT positive and negative reactions. A RT-negative control reaction was carried out for each sample in which the superscript enzyme was replaced by taq polymerase. All PCR reactions were resolved on 2.0% agarose gels. 

For each transgenic mosquito line, RNA was harvested from 20 mosquitoes G_3_ by homogenization in 500 µL of trizol, centrifugation for 10 min at 12,000 g at 4 °C, extraction of supernatants with 100 µL of chloroform per 500 µL of trizol, and precipitation from the aqueous fraction with 250 µL of isopropanol and centrifugation for 10 min at 4 °C, 12,000 g. RNA pellets were washed with 70% ethanol, resuspended in distilled water, and the concentrations determined spectrometrically. A total of 7.5 µg of each RNA sample was Turbo DNase I (Ambion, Austin, TX, USA) treated, followed by inactivation of DNases at 65 °C for 15 min. Equal volumes of DNase treated RNAs were utilized to perform RT-positive, RT-negative, and control beta-actin reactions using Superscript III one step RT-PCR (Invitrogen, USA). The forward primer, Actin 2f: atggtcggtatgggacagaaggactc and reverse primer, Actin 8R: gattccatacccaggaaggaagg were utilized for the beta-actin control reaction [27].

### 2.5. TCID_50_ Immunofluorescence Assays

Supernatants collected at 3 dpi were serially 10-fold diluted into wells of a 96-well plate, and Vero cells were added to each well at a density of 2 × 10⁴/well. Following three days incubation the cells were fixed with 3:1 mixture of acetone:Dulbecco’s Phosphate Buffered Saline (DPBS) for 30 min and then stained with 1:100 dilution of monotypic primary antibody against CHIKV capsid protein (Virostat, Westbrook, ME, USA) for 40 min, followed by a mouse-specific biotinylated secondary antibody (G.E. Healthcare) and a detection system of FITC-conjugated streptavidin (Invitrogen, Grand Island, NY, USA) tertiary antibody. Wells exhibiting cytoplasmic fluorescence were scored as positive. Virus titers were calculated based on Karber’s method [28]. 

### 2.6. Caspase 3 Assays

Caspase 3 activity was measured using the caspase-glo 3/7 assay (Promega) according to the manufacturer’s instructions. Vero cells were plated at 2 × 10^3^/well in 96-well plates 24 h before infection. These indicator cells were infected with 3 dpi supernatants collected from the hRz transformed clonal cell lines that displayed complete suppression in the TCID_50_-IFA assays. We included an uninfected cell control to determine the background rate of apoptosis for our culture conditions, as well as a control to account for luminescence generated by the media. At 2 dpi the cells were incubated with caspase glo reagent for one hour in the dark at room temperature. Caspase 3 activity was quantitated by measuring luminescence using a LMAX-2 luminometer (Molecular Devices, Sunnyvale, CA, USA). 

### 2.7. Quantitative RT-PCR

Viral RNA was extracted at 3 dpi from the hRz clonals exhibiting complete suppression by TCID_50_-IFA using a Mini Viral RNA kit (Qiagen, Valencia, CA, USA). Control CHIKV with known titer was analyzed to generate the standard curve. CHIKV genomic RNA was copied using the Gene Amp RNA PCR MULV Reverse Transcriptase Kit (Applied Biosystem, Grand Island, NY, USA) using a single primer targeting the nsP2 reverse primer, aaattcggcctgaaccttct [29] and one cycle of 30 min at 42 °C and 5 min at 99 °C. Quantitation was performed using the 7500 Fast Real Time PCR System (Applied Biosystem) with one cycle of 2 min at 50 °C, one cycle of 10 min at 95 °C, and 40 cycles of 15 s at 95 °C and 1 min at 60 °C. Syber Green detector was used, and a dissociation curve analysis was incorporated into each cycle with data collected from step 2 of stage 3 of the cycle. 

Results were analyzed using the ABI Sequence Detection Software Version 1.3. For quantitation, a second primer set, nsP2 forward primer ttctgggggtcagagaaaga and nsP2 reverse primer aaattcggcctgaaccttct [29], was used. The appropriate concentrations of the primer (20 pmol/µL) were determined by examining the efficiency of the standard curve (the slope of −2.8 and r^2^ value of 0.97 shows 100% amplification). Power Syber Green PCR master mix (Applied Biosystem) was used for all qRT-PCR reactions. The absolute quantitation of CHIKVviral RNA copies per mL in the sample were determined by comparing them with standards of known viral titer.

### 2.8. Generation of hRz Transgenic Mosquito Lines

A total of 660 and 731 G_0_ embryos were injected with either *piggyBac* vector 3XP3-ECFP + t-RNA^val^ Pol III + hRz #9 + poly A_60_ or 3XP3-ECFP + t-RNA^val^ Pol III + hRz #14 + poly A_60_ constructs, respectively, along with the phspBAC helper plasmid [30] (ITF insectary, University of Maryland, College Park, MD, USA). The G_0_ embryos were hatched at three days post-injection in deoxygenated water, transferred to de-chlorinated water, and fed with liver powder. Larvae were maintained at 26 °C, 80% humidity with a 12 h light/dark cycle and outcrossed with wild-type HWE Resulting G_1_ larvae were screened at the fourth instar for eye fluorescence, and positive individuals were outcrossed for two generations followed by inbreeding. A total of nine separate lines were generated, two expressing hRz #14 and seven expressing hRz #9. 

### 2.9. Splinkerette PCR

Genomic DNA was isolated from six transgenic females of each line using a genomic DNA purification kit (Promega) and the manufacturer’s protocol with minor changes. The spPCR protocol was adopted from Potter and Luo [31] using 1 µg of genomic DNA digested overnight for each of four different restriction enzymes (BamHI, BglII, BstYI and BfuCI), followed by ethanol precipitation of the digestion reaction. The SPLNK oligonucleotide was prepared by annealing 150 ng/µL each of the SPLNK-GATC-TOP (gatcccactagtgtcgacaccagtctctaattttttttttcaaaaaaa) and SPLNK-BOT (cgaagagtaaccgttgctaggagagaccgtggctgaatgagactggtgtcgacactagtgg) oligonucleotides [31] in 100 µL of 10X NEB buffer 2 and 800 µL of water at 95 °C for 3 min followed by cooling to room temperature. The purified digestion reactions for each restriction enzyme were then ligated with SPLNK oligonucleotide at room temperature overnight. 

The ligation reaction was heat inactivated for 10 min at 65 °C followed by two rounds of nested PCR reactions. The PCR reaction one used SPLNK #1 primer, cgaagagtaaccgttgctaggagagacc, and 5’ SPLNK-PB#1, accgcattgacaagcacg. PCR reaction two used 0.5 µL of product from reaction 1 with SPLNK #2, gtggctgaatgagactggtgtcgac, and 5’SPLNK-PB#2, ctccaagcggcgactgag [31]. PCR reaction two was resolved on a 2% gel, followed by gel purification of all the bands (Promega, wizard SV Gel and PCR clean up system) and sequencing using 5’SPLNK-PB#2 primer. The sequences were searched for the characteristic 5’-LTR sequence of the *piggyBac* vector, catgcgtcaattttacgcagactatctttctaggg, having an associated TTAA insertion site downstream, followed by mosquito genomic sequences using BioEdit (Softpedia, Carlsbad, CA, USA). The mosquito sequences were used to search the VectorBase *Ae. aegypti* genome database [32] to identify the location of the transgene insertions.

### 2.10. Mosquitoes, Infection and Maintenance

The CHIKV 181/25 strain [33] was used for all mosquito challenges. A fresh viral inoculum was prepared prior to each feeding by inoculating T-75 flasks of 24-h-old Vero cell cultures with an MOI of 2 in serum free DMEM with 2 h of gradual shaking at 37 °C. Following inoculation 7 mL of DMEM with 10% FBS was added and the cultures incubated at 37 °C for two days before harvesting infectious supernatants for mosquito blood meals. 

Infectious supernatants were mixed with equal volumes of citrated blood (Colorado Serum Company, Denver, CO, USA), and 100 µL of ATP (1 mM)/10 mL and 0.8 mL of 20% sucrose/mL were added as a phagostimulants [34]. A non-infectious blood meal derived from uninfected Vero cell culture supernatants was used to feed negative control mosquitoes. Ten-day-old adult female mosquitoes were deprived of sucrose for 72 h and water for 12 h before blood feeding [33]. 

All mosquito infections were performed in a BSL-2 facility as CHIKV strain 181/25 is a RG2 agent according to NIH (National Institutes of Health and CDC (Centers for Disease Control and Prevention guidelines [35,36]. A glass membrane feeding apparatus heated at 40 °C using a re-circulating water bath was filled with a sufficient volume of blood meal to cover the entire membrane and mosquitoes were allowed to feed for 1 h. Both pre and post blood meal samples were collected from the feeders to calculate the viral titers over the feeding period. Following feeding the mosquitoes were briefly anesthetized using carbon dioxide and fully engorged females were sorted and transferred into rearing cages and maintained at 26 °C, 80% relative humidity with a 12 h light/dark cycle for seven days. At seven dpi both control and experimental mosquitoes were individually collected in 200 mL of DMEM, homogenized, and filtered using 0.20 µM syringe filters (Fisher-Brand) for TCID_50_-IFA analysis.

### 2.11. Genotyping of Mosquitoes

A set of primers was designed against the native insertion site sequences for each transgenic mosquito line based on the splinkerette analysis (Supplementary Table S2). Genomic DNA was isolated from individual G_6_ mosquitoes of each line using a genomic DNA purification kit (Promega), and 25 µg of genomic DNA was analyzed by direct PCR using the One Taq Quick Load master mix (NEB) and mosquito-specific primers that amplify across the identified native transposon insertion site in each transgenic line. The PCR reactions were resolved on 2% gels, and the presence or absence of the predicted native fragment was used to distinguish between heterozygous (PCR-positive) and homozygous (PCR-negative) individuals. 

### 2.12. In Situ Indirect Immuno-Fluorescent Assays

Mosquito heads and midguts were dissected in phosphate buffer saline (PBS) from both experimental and control mosquitoes at seven days post-feeding on CHIKV-infected blood [37]. Head squashes or midguts were fixed in cold acetone at −20 °C for 15 min, dried, and stained with primary antibody specific to CHIKV capsid protein (Virostat) for 40 min at 37 °C. The slides were then washed with PBS 2X for 2 min each, dried, and Alexa flour-conjugated secondary antibody (Invitrogen) was used to stain at 37 °C for 40 min followed by a wash with PBS and another single wash with deionized water. The slides were allowed to dry for 25 min under the hood and then mounted with glycerin: PBS (9:1). 

### 2.13. Assays of Virus in Saliva

Control and G_7_ transgenic lines BM16, BM8, BM2 and CM10 were feed infectious blood meals with a viral titer of 3 × 10^9^ TCID_50_/mL. Both the infected controls and hRz transgenics were maintained for seven days, and then allowed to probe and feed on 700 µL probing solution (50% FBS (164 mM) + NaCl (100 mM) + NaHCO_3_ (0.2 mM) + ATP (50 µg) + sucrose, pH 7.0) for 2 h. Virus titers were determined following probing by TCID_50_-IFA on both the probing solution and the mosquito homogenates [38].

## 3. Results

### 3.1. Construction of Transformation Vectors Carrying Anti-CHIKV hRz

**Figure 1:**
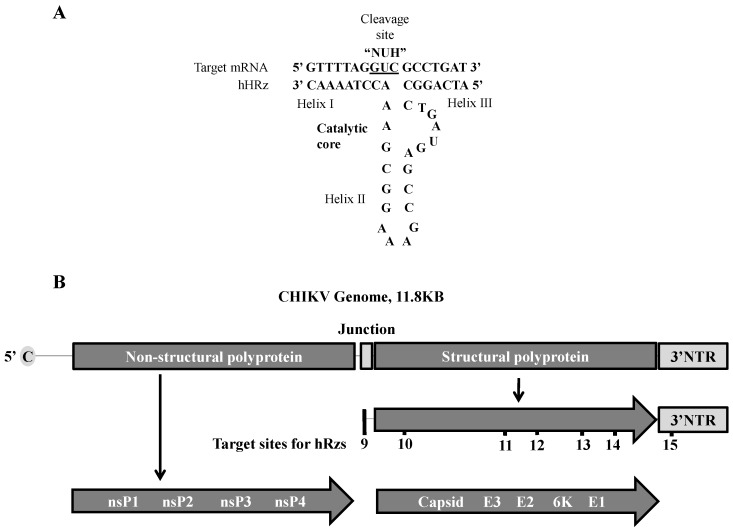
Generalized structure of a minimal hammerhead ribozyme (hRz) and organization of the chikungunya virus (CHIKV) viral genome showing hRz target sites. (**A**) hRzs consist of helix I and III which mediate complementary base-pairing with the target, and helix II which acts as the catalytic core mediating cleavage of the target RNA; (**B**) Schematic of the CHIKV genome. nsP1–nsP4 = polyprotein encoded by the genomic RNA. Capsid, E3, E2, 6K and E1 = polyprotein encoded by the sub-genomic RNA. 9–15 = target sites for hRz#9-#15.

hRzs are small catalytic RNAs that mediate a sequence-specific cleavage reaction with a complimentary target RNA molecule. Complimentary base-pairing with the target RNA forms helix regions, helix I and helix III, that flank a catalytic core within helix II (Figure 1A). Cleavage of the target RNA occurs at the 3’ end of a NUH triplet within the target RNA sequence, where N can be any nucleotide, H can be any nucleotide except G, and U is Uracil. While several different triplet-flanking sequence combinations can be cleaved, the kinetics of the cleavage reactions are best using GUC as this triplet is found most frequently among the natural targets of hRzs [39]. We therefore designed several hRzs based upon ClustalX alignment studies of 100 different CHIKV strains available in the GenBank sequence database searching for 18–19 bp conserved genomic sequences that included a GUC cleavage site between nt 8 and 12 of the sequence. The cleavage activity of each ribozyme was confirmed against an artificial target RNA using an *in vitro* cleavage assay as previously described [25].

Seven highly conserved 18–19 bp sequences with the most preferred catalytic core triplet “GUC” were identified in the structural protein region of the viral genome (Figure 1B) which plays a vital role in CHIKV packaging. hRz #9 targets the internal promoter for the subgenomic mRNA; hRz #10 targets the capsid protein gene; hRz #11 and #12 target the envelope protein gene E2; hRz #13 and #14 target the envelope protein gene E1; and hRz #15 targets the 3’NTR which is responsible for the stability of viral mRNAs.

Using specific PCR primers (see Materials and Methods), we cloned the *Ae. aegypti* t-RNA^val^ Pol III promoter upstream and added a poly A_(60)_ tail downstream of each hRz [25]. Linking each hRz to the t-RNA^val^ sequence provides an internal RNA Pol III promoter that increases the level of expression. Additionally, the t-RNA^val^ Pol III sequence makes the fusion RNA resistant to RNA nucleases and helps in co-localizing the hRz with the target sequence in the cytoplasm [25,40]. The addition of a poly A_(60)_ tail recruits RNA helicases, which helps in increasing the accessibility of the target sequence for the hRz [25,41]. The entire expression cassette was cloned into a hygromycin selectable transformation vector pLAeRz#ARH [25], followed by the cloning of a CMV-driven DsRed marker gene for efficient selection of the transformed selected cell line [25].

### 3.2. Single-Cell Sorting for Transformed Selected Cell Populations and Screening for CHIKV Suppression

To insure that we demonstrated the optimal effectiveness of a given hRz, we examined CHIKV suppression in clones derived from transformed selected cells. Vero cells transformed with each hRz were first selected on hygromycin (200 µg/mL) for three to four weeks and then sorted as single cells into a 96-well plate using the DsRed fluorescent marker as an indicator of cell transformation. The sorting analysis showed that only a percentage of selected cells (30%–50%) were actually transformed (Supplementary Figure S2). 

**Figure 2:**
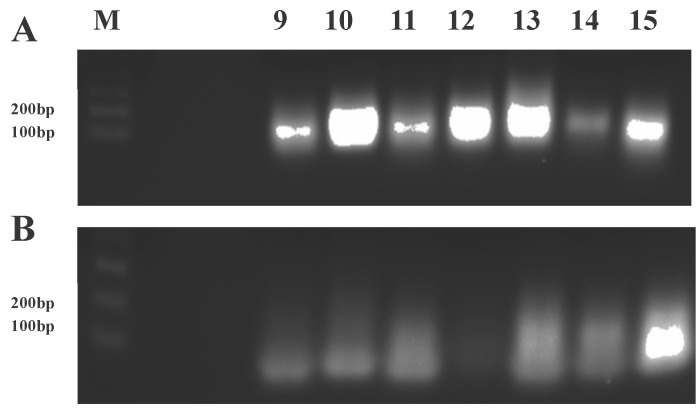
Real-time polymerase chain reaction (RT-PCR) analysis of transformed Vero cells. The presence of a 130 bp fragment (t-RNA^val^ promoter + ribozyme) for each ribozyme (**A**) demonstrates stable transformation of the clonal cells. The absence of this 130 bp fragment in the RT-negative control (**B**) indicates a lack of genomic DNA contamination in the isolated RNA. The low molecular weight smears apparent in each lane are attributed to primer dimers as they are well below the lowest size standard in the gel. The forward primer used was specific to the common t-RNA^val^ promoter, and the reverse primer was specific for each ribozyme. M = 1 kb plus DNA ladder, lanes 9–15 correspond to hRzs #9 through #15.

Cellular expression of hRz in each clonal cell population was confirmed by performing RT-PCR on purified total cellular RNA using a common t-RNA^val^ forward primer and a hRz-specific reverse primer (Supplementary Table S1). The presence of a desired product of 130 bp in 2% agarose gels confirmed the cellular expression of each hRz (Figure 2).

**Figure 3:**
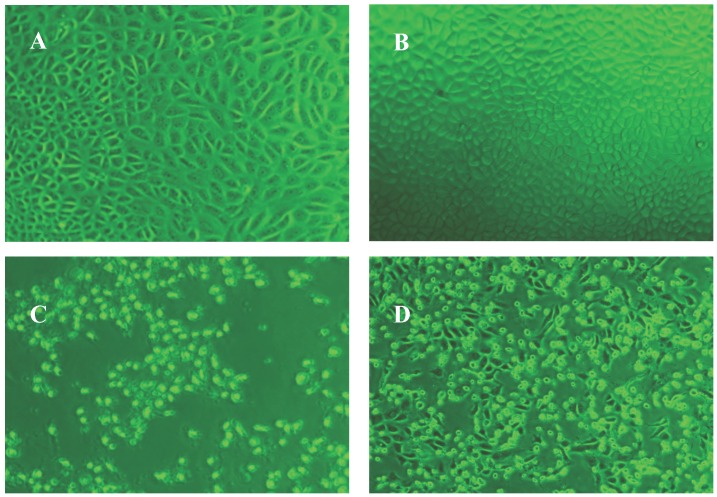
Comparative characteristic cytopathic effect (CPE) of wild types (day 3) and clonal populations (day 7). Images were taken at 40× magnification. (**A**) hRz #9 and (**B**) hRz #14 expressing cells with no CPE where (**C**) and (**D**) are untransformed Vero cells showing severe CPE.

For each hRz construct, a total of 24 clones were challenged with CHIKV 181/25 at an MOI of 0.005. After incubating for seven days at 37 °C, the challenged clones were screened based upon the absence of a characteristic CHIKV CPE compared to the untransformed wild-type Vero cells (Figure 3). Clones lacking visible CPE were further analyzed by TCID_50_-IFA to confirm the absence of CHIKV replication. 

### 3.3. TCID_50_-IFA Analysis of CHIKV Suppression in Clones Lacking Visible CPE

**Figure 4:**
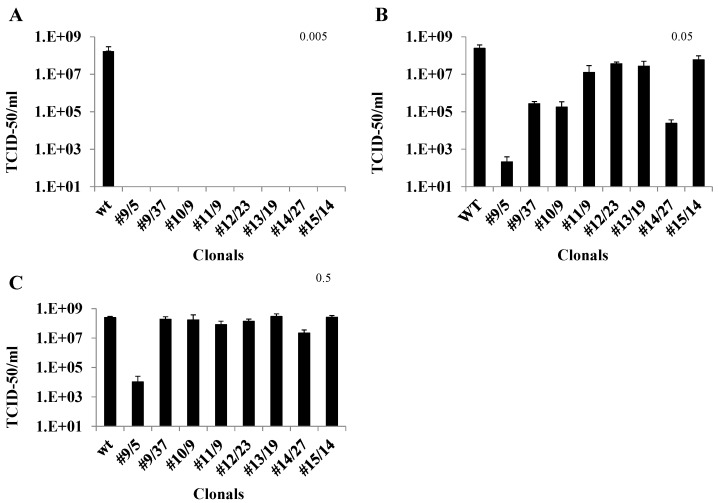
TCID_50_-IFA analysis of transformed Vero cell clones challenged with three different multiplicity of infections (MOIs) of CHIKV. (**A**) Challenge MOI of 0.005, and stained with anti-CHIKV antibody at 3 dpi; (**B**) Challenge MOI of 0.05, analyzed at 2 dpi. (**C**) Challenge MOI of 0.5, analyzed at 2 dpi. Each bar represents an average CHIKV titer obtained from three independent infections. Statistically significant differences were determined relative to the infected control sample (wt) using one-way ANOVA (GraphPad prism 6.0 software, Dunnett’s, *p* < 0.01). Error bars show the standard deviation among the three replicates for each clone. Individual clones are designated as hRz number/clone number.

Both untransformed Vero cells and hRz expressing cloned cells were plated at a density of 2 × 10^5^ cells/well 24 h before infection. The TCID_50_-IFA analysis of CHIKV challenges of clonal transformed cell populations demonstrated complete suppression at MOI 0.005 for all hRzs (Figure 4A). While these results were promising, they did not allow a determination of the relative effectiveness of a given hRz compared to the others. A more effective hRz should exhibit relatively greater suppressive capability at higher MOIs where complete suppression is not possible. We therefore analyzed hRz-transformed Vero cell clones at MOIs of 0.05 and 0.5 to attempt to identify the most effective hRzs.

TCID_50_-IFA analysis of hRz-expressing clones challenged with CHIKV at an MOI of 0.05 revealed differences in the relative suppressive capabilities among the hRz clones (Figure 4B). The most effective suppression was observed with hRz clones #9/5, #9/37, #14/27 and #10/2, which ranged from six, three, four and four orders of magnitude in reduction of virus productivity, respectively (Figure 4B). These results revealed that variability was evident even among clones expressing identical hRz constructs, likely due to position effects related to the hRz transgene insertions. Infections at 0.5 MOI demonstrated that clone #9/5 was 5.5 orders of magnitude less productive compared to untransformed Vero cells and clone #14/27 still exhibited one order of magnitude of suppression (Figure 4C). Based upon these data the two most effective hRzs in our screen appeared to be #9 and #14.

### 3.4. qRT-PCR Analysis of Ribozyme-Expressing Cell Clones Demonstrates Complete Suppression

**Figure 5:**
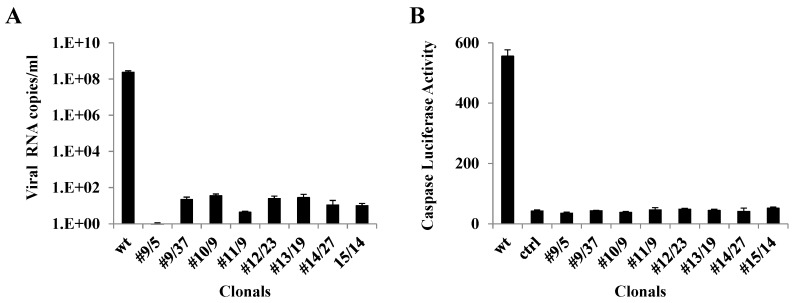
Real-time PCR and caspase 3 activity of CHIKV-challenged transformed Vero cell clones. (**A**) Viral RNA was obtained from 3 dpi cell culture supernatants challenged at an MOI of 0.005. Quantification was performed using a primer specific to the nsP2 region of the CHIKV (Materials and Methods). (**B**) Supernatants collected from 3 dpi cell cultures challenged at an MOI of 0.005 were used to infect untransformed Vero cells, and caspase 3 activity was measured at 2 dpi using a standard luciferase activity (Materials and Methods). Uninfected cell controls (ctrl) were also used to screen for background caspase 3 activity. Statistically significant differences (Dunnett’s, *p* < 0.01) relative to the infected control sample (wt) were determined using one-way ANOVA (GraphPad prism 6.0 software).

The TCID_50_-IFA results obtained from infection of clonal Vero cell isolates at 0.005 MOI were validated by qRT-PCR (Materials and Methods) using the same supernatants from 3 dpi. The primer employed was specific to the nsP2 protein region of the virus genome. The results of the qRT-PCR complemented the TCID_50_-IFA analysis (Figure 5A). Significant levels of viral RNA amplification were obtained with untransformed wild-type Vero cells compared to minimal or undetectable viral RNA amplification in the case of clonal cell populations expressing the hRz. 

### 3.5. Caspase 3 Assays of Ribozyme-Expressing Cell Clones Confirms Lack of CHIKV Infection

Previous studies have demonstrated that alphaviruses employ apoptosis [42,43] as a means to more effectively spread infection in the host cell, while at the same time escaping the host immune response [44]. Therefore, measuring the amount of apoptosis that a cell culture exhibits following challenge with CHIKV can be an effective means of determining the effectiveness of our hRz in suppressing CHIKV infection. Since CHIKV employs both extrinsic and intrinsic pathways in order to induce apoptosis, measuring the level of caspase 3 can easily assess the effectiveness of each hRz. Caspase 3 levels were determined using the 3 dpi supernatants collected from the 0.005 MOI suppression challenge experiment for clones (Materials and Methods). None of the hRz clones analyzed exhibited a significant level of caspase 3 activity, indicating these cells were healthy following the CHIKV challenge (Figure 5B).

### 3.6. Construction of *PiggyBac* Vectors and Establishment of Transgenic Mosquitoes

The tRNA^val^ Pol III hRz #9 + poly A_60_ and tRNA^val^ Pol III hRz #14 + poly A_60_ sequences were PCR-amplified from pLAeRz#9RH and pLAeRz#14RH, respectively, using a common set of forward and reverse primers (Material and Methods). These amplified 200 bp fragments were then cloned in the pXL-BacII-3xP3-ECFP *piggyBac* vector plasmid [26]. A total of 660 and 731 preblastoderm embryos were injected with pXL-BacII-3xP3-ECFP-tRNA^val^ Pol III hRz # 9+poly A_60_ and pXL-BacII-3xP3-ECFP-tRNA^val^ Pol III hRz # 14+poly A_60_, respectively, along with a helper plasmid phspBAC [30]. A total of 357 and 317 G_0_ embryos were hatched, respectively, for #9 and #14 (Supplementary Table S3). 

G_0_ adults were batch-mated with HWE mosquitoes, leading to G_1_ larvae, which were screened for the eye-specific cyan fluorescent protein. The transformation efficiency for G_1_ was anywhere from 0.1% to 1.6% (Supplementary Table S4). We selected a total of seven and two different lines expressing hRz # 9 and hRz #14, respectively, for further analysis based on strong eye-specific fluorescence. G_1_ adults were then outcrossed for two more generations followed by inbreeding until analysis. Approximately 50%–60% of G_2_ larvae were positive for eye fluorescence in all lines except BM16, which exhibited 75% positives. At G_6_, the percentage fluorescence was anywhere from 60%–80%.

### 3.7. RT-PCR to Confirm Transgene Expression

**Figure 6:**
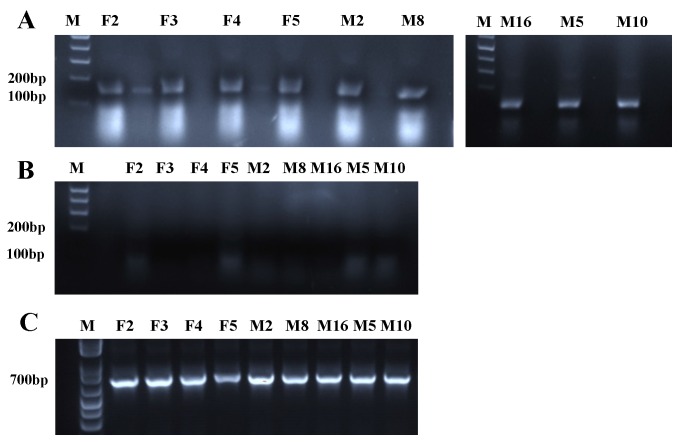
RT-PCR of transgenic mosquitoes. (**A**) Gel displays the presence of a 130 bp fragment, confirming the expression of hRzs in each transgenic mosquito line. (**B**) RT-negative control reactions display the absence of the 130 bp fragment, confirming a lack of DNA contamination. (**C**) Gel displays a 700 bp fragment corresponding to the beta-actin internal control for each line. M = 1 kb plus DNA ladder. F2, F3, F4, F5, M2, M8 and M16 are transgenic mosquito lines expressing hRz #9, and M5 and M10 express hRz #14.

A total of 20 mosquitoes were trizol-extracted for each line (Material and Methods). For each line, 7.5 µg of RNA was DNAse-treated and an equal volume of the RNA was used for an RT-positive, an RT-negative, and a control reaction for the beta-actin housekeeping gene using the Superscript III one-step RT-PCR kit (Invitrogen). The presence of a 130 bp band in the RT-positive reactions confirmed the stable expression of the transgene for each line. No amplified product was observed in the RT-negative controls, confirming a lack of DNA contamination. The 700 bp beta-actin control band was also evident for all nine lines (Figure 6).

### 3.8. Identification of Each Transgene Integration Site via Splinkerette PCR

Utilizing the protocol of Potter and Luo [31], genomic DNA was isolated from six female mosquitoes for each line at G_2_. A total of 1 µg of each genomic DNA was digested with BamHI, BglII, BstYI and BfuCI, followed by purification with precipitation. The purified DNA for each enzymatic digestion was then ligated with the SPLNK oligonucleotide, followed by two sets of nested PCR reactions (Materials and Methods). The second PCR reaction product was resolved on 2% gels and the bands for each reaction were gel-purified followed by sequencing. The sequencing data was searched for the *piggyBac* vector specific 5’ LTR sequence and TTAA insertion site, and the supercontig numbers corresponding to the transgene insertion were identified using VectorBase *Ae. aegypti* online software [32]. The analysis showed that each of the nine lines exhibited different insertion sites in the *Ae. aegypti* genome (Supplementary Tables S5 and S6). 

### 3.9. Analysis of CHIKV Infection Suppression in hRz Transgenic Mosquitoes

**Figure 7:**
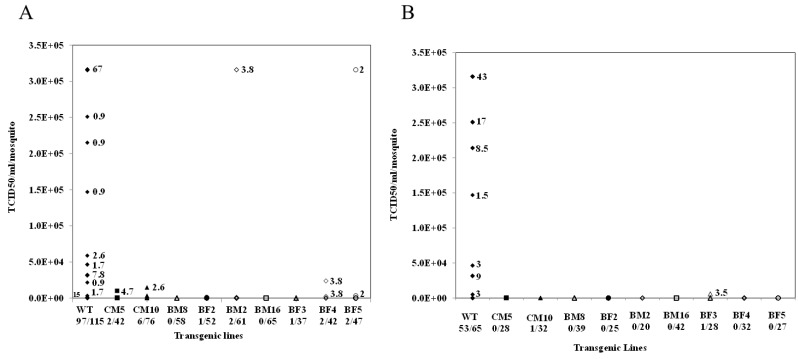
TCID_50_-IFA performed on CHIKV challenged transgenic mosquito lines. (**A**) Results of TCID_50_-IFA performed on partially heterozygous G_6_ compared to non-transgenic HWE controls (WT). (**B**) Results of TCID_50_-IFA performed on individual blood-fed heterozygous female mosquitoes of the G_9_ compared with Higgs White Eye (HWE) (WT) controls. All titrations were at seven days post blood meal. The percentage of mosquitoes exhibiting a particular viral titer is presented in the graph. The actual number of infected mosquitoes along with the total number of blood-fed female mosquitoes analyzed for each line and the WT control are presented below each X-axis designation.

Ten-day-old transgenic females selected based upon eye-specific fluorescence and HWE control mosquitoes were starved of sucrose and water for 72 h and 12 h, respectively. An artificial infectious blood meal was prepared with a 1:1 ratio of infectious cell culture supernatant and citrated blood with added ATP and sucrose. The mosquitoes were blood-fed for one hour followed by incubation for seven days at 25 °C and 80% relative humidity. TCID_50_-IFA analyses were performed on whole body trituration of each blood-fed mosquito for all nine transgenic lines, as well as HWE control mosquitoes. Two of the transgenic lines, BM16 and BM8, demonstrated undetectable CHIKV titers for all 65 and 58, respectively, mosquitoes analyzed in each line. Transgenic lines CM5, BF2 and BF3 yielded two, one and one infected mosquitoes out of 42, 52, and 37 total blood-fed females, respectively. For the remaining transgenic lines, BF5, BF4, BM2 and CM10, there were a few CHIKV-positive individuals among the blood-fed females, but in all cases the viral titers were reduced two to three orders of magnitude compared with those obtained for the HWE control mosquitoes (Figure 7A).

To further establish the efficacy of our transgenic lines, we backcrossed G_8_ individuals of all transgenic lines with wild-type HWE mosquitoes. Female G_9_ heterozygous transgenic mosquitoes were then infected by feeding CHIKV 181/25 and were then analyzed using TCID_50_-IFA. Seven transgenic lines, BM16, BM8, BF4, BF5, BM2, BF2 and CM5, exhibited complete resistance to CHIKV infection. Two lines, BF3 and CM10, had a single infected mosquito each out of a total of 16 and 23 blood-fed females analyzed, respectively, with viral titers three orders of magnitude lower than the wild-type control mosquitoes (Figure 7B). RT-PCR analyses and sequencing demonstrated that the virus from these infected mosquitoes retained an intact target site, eliminating the possibility of escape mutations having led to CHIKV infection in our transgenics.

**Figure 8:**
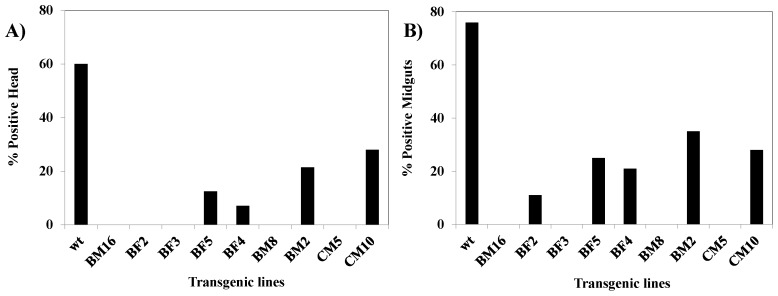
Indirect immunofluorescent assay preformed on G_7_ transgenic females challenged with CHIKV. Seven dpi heads and midguts were dissected from both HWE and transgenic lines. (**A**) Shows the percentage of IFA-positive head squashes; (**B**) Percentage of IFA-positive midguts.

In a separate analysis, we infected multiple females from each transgenic line as well as the control HWE strain and performed IFA on head squashes and midguts as previously described [37]. Viral infection was absent in most transgenic lines, with only a few individuals exhibiting head fluorescence in the BM2 and CM10 lines. All control HWE individuals exhibited fluorescence (Figure 8). This observation complemented the TCID_50_-IFA results.

### 3.10. Direct PCR Analysis Confirms Haploid Genotype of G_6_ mosquitoes

Direct PCR analysis was performed on five of the transgenic lines that gave minimal levels of CHIKV infection, BM16, BM8, CM5, BF2, and BF3. Sets of forward and reverse primers were designed complementary to the genomic DNA supercontigs specific for each transgenic line (Supplementary Table S2). Direct PCRs of genomic DNAs for each line were resolved on 2% gels, and the presence of the predicted amplified bands confirmed the transgenic lines were each predominantly heterozygous for their hRz antiviral gene. For transgenic line BM8, all 18 of the tested DNA samples were positive for PCR product, confirming a heterozygous genotype for the mosquito populations. The percent homozygosity for each line is shown in (Supplementary Table S7). 

### 3.11. CHIKV Titers in Saliva Collected from Challenged Transgenic Female Mosquitoes

**Figure 9:**
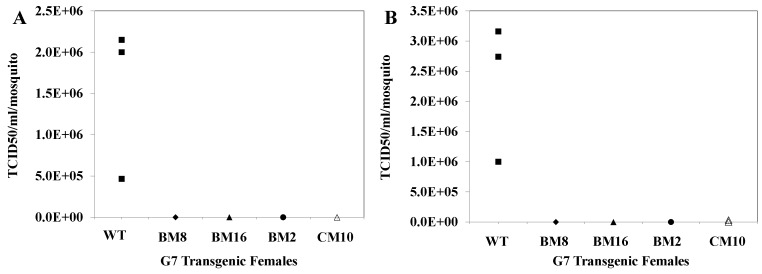
Saliva titers performed on G_7_ transgenic mosquitoes challenged with CHIKV. Three pools of 15 female mosquitoes were collected at seven days post blood feeding for each transgenic line and were allowed to probe a feeding solution for 2 h. TCID_50_-IFA was performed on both the feeding solution (**A**) and whole body homogenates (**B**) of the mosquito pools. None of the transgenic lines tested exhibited significant viral titers compared with the control (WT).

We also examined the possibility that some CHIKV might be detected in the saliva of infected transgenic mosquitoes. Transmission assays were performed on two lines that exhibited complete suppression of virus infection, BM8 and BM16, and two that demonstrated some individuals that supported low levels of infection, BM2 and CM10. Fifteen transgenic females from each of these lines, along with the HWE control, were maintained for seven days following an infectious blood meal. Following this incubation period, all 15 individuals from each line were fed on a probe solution for one hour. TCID_50_-IFA performed on the pooled probe solution collected from HWE control mosquitoes demonstrated an average viral titer of 1.5 × 10^6^ TCID_50_/mL, while no virus was evident in the probe solutions collected from BM8, BM16 and BM2, and a viral titer of 7 × 10^1^ TCID_50_/mL was obtained for the CM10 pool (Figure 9). Similarly TCID_50_-IFA performed on the whole body trituration of 15 control mosquitoes/cage yielded an average viral titer of 2.3 × 10^6^ TCID_50_/mL while no virus was evident with the BM16, BM8 and BM2 transgenic lines, and a slight viral titer of 1.0 × 10^4^ TCID_50_/mL was present for CM10. These results demonstrate that hRz antiviral catalytic activity was capable of completely inhibiting the transmission potential of CHIKV in the natural mosquito vector.

## 4. Discussion

Our results demonstrate that hRzs can be extremely effective against CHIKV not only in infected cell cultures, but also as transgenic antivirals in mosquitoes. Our analyses followed a systematic evaluation of each hRz construct from *in vitro* cleavage assays through suppression of infections in transformed clonal cell cultures, and in heterozygous transgenic mosquitoes. 

We used Vero cells for our analyses because these cells are highly susceptible to CHIKV and support its rapid proliferation [45]. They also yield a prominent CPE [46] that facilitates preliminary screening of clones for effective suppression of virus replication. The CHIKV 181/25 strain [33] used in our suppression assays is as efficient in Vero cell culture as the wild-type LR CHIKV strain. The choice of Veros as the experimental cell line coupled with the CHIKV 181/25 strain of the virus makes our experimental system more stringent in establishing the efficacy of each hRz.

Several factors must be optimized for these hRzs to have efficient suppressive activity against a replicating virus in infected cells. Since there will be an abundance of viral genomic RNA produced in the cytoplasm during replication, successful suppression requires a robust transcription rate, co-localization of the ribozyme to the same cellular compartment(s) as the target molecule, and an effective ribozyme-target interaction. *Trans*-acting ribozymes have been described that allow optimal cleavage *in vivo* when these critical facts are considered. Fusion of the *Ae. ageypti*–derived t-RNA^val^ Pol III promoter upstream of the hRz allows efficient expression of the transgene and assists in the co-localization of the ribozyme with the target RNA in the cytoplasm [25,26,27,28,29,30,31,32,33,34,35,36,37,38,39,40]. A polyA_(60)_ tail fused downstream of the hRz promotes recruitment of RNA helicases that remove secondary structures in the RNA target and enhance accessibility of the target site [25,41].

For cell culture transformations we cloned each hRz construct into a transformation vector, pLAeRz #ARH, containing an RSV-promoted hygromycin gene [25]. Transfected Vero cells selected with 200 µg/mL hygromycin yielded enriched populations of transformed cells which were then sorted to establish clonal populations of all seven hRzs. Infection of these clonals with greater MOIs of 0.05 and 0.5 demonstrated that hRz #9/5 was the most effective clone, exhibiting 5.5–6 orders of magnitude greater suppression, followed by clones #14/27 and #10/2 (Figure 4). The observed variation among clonal populations expressing a given hRz suppressor gene is not uncommon [47,48] and is likely attributable to position effects on transgene expression. However, considering the number of individual clones analyzed for each hRz, it is more likely that these differences are reflective of the relative effectiveness of each ribozyme against its target site in the virus genome.

Since CHIKV induces apoptosis in host cells, the caspase 3 assay is a convenient measure of apoptotic activity [44]. If a given hRz is effective in suppressing CHIKV infection, there will be less caspase 3 activity upon CHIKV challenge of cells expressing the hRz compared to untransformed Vero cells. Our results confirm there is no detectable caspase 3 activity in cell clones expressing the hRzs upon challenge with 0.005 MOI of CHIKV (Figure 5B).

Similarly, qRT-PCR analysis of these same clones displayed negligible amplification of the CHIKV genome sequences compared with the untransformed wild-type Vero cells (Figure 5A). The small level of amplification detected in some clones may be due to the presence of defective virus particles that may result from effects on packaging of the viral genomes and/or assembly and release of virions due to genomic RNA degradation. In contrast, both the TCID_50_-IFA and caspase-3 assays quantify the infectious potential of a viral inoculum and therefore do not detect defective particles or genomes.

These infected cell analyses demonstrate that appropriately configured hRzs can be effective in suppressing CHIKV infection at physiologically relevant virus titers. While our model system of Vero cells and the CHIKV 181/25 strain demonstrates that at higher MOIs, the virus seems to outpace the levels at which our hRzs can be expressed, in the case of mosquito cells and natural virus isolates, virus replication is far less robust [49]. These results allowed us to select hRz #9 and #14 as our most effective hRzs for inhibiting CHIKV replication and transmission in transgenic mosquitoes. 

The multiple turnover kinetics of hRzs favors efficient cleavage of viral RNA, allowing a single hRz molecule to cleave multiple viral RNAs [50]. Moreover, the gene silencing induced by RNAi is sometimes reversible and at times may lead to transient suppression [51], in which the function of target molecules is not destroyed [52]. In contrast, hRzs act to cleave the target viral RNA irreversibly, providing a reliable and stable approach to inhibition of viral infection in host cells. In addition, since the target region length required for hRzs to act is only 12–19 nt long, this could decrease the potential for the development of escape mutants.

We also confirmed the effectiveness of hRz #9 and #14 as CHIKV antivirals in transgenic *Ae. aegypti* mosquitoes. The *piggyBac* transposon was employed for this purpose because it provides a relatively high rate of stable integration and does not exhibit post-integration remobilization in *Ae. aegypti* [53], allowing us to recover several unique transgenic lines having independent insertion events from a single round of embryo injections. A total of nine transgenic lines were established and confirmed as having unique insertions within the mosquito genome by splinkerette PCR (Supplementary Table S5). Expression of the transgene in each line was confirmed through RT-PCR compared with a control reaction for the beta-actin housekeeping gene (Figure 6). 

We confirmed previous observations that viral titers reach a maximum by day seven post blood feeding in *Ae. aegypti* [54], although a few blood-fed females exhibited a lower viral titer compared to the maximal titers of many control HWE mosquitoes. This could be due to the physiological state of individual mosquitoes during blood feeding. Starving the mosquitoes for 72 h before an infectious blood meal may result in stress that leads to less favorable feeding behavior. While similar individuals are likely present among the transgenic lines, the lack of a significant number of individuals attaining maximal virus titers indicates effective virus suppression. 

TCID_50_-IFA analysis of whole body extracts at G_6_ confirmed that two of our lines, BM16 and BM8, completely inhibit CHIKV production. Transgenic lines CM5, BF2 and BF3 yielded only one or two infected mosquitoes while the other four lines had only a few infected mosquitoes. In contrast, the untransformed control HWE mosquitoes yielded viral titers of 3 × 10^5^ TCID_50_/mL in nearly 67% of the infected mosquitoes (Figure 7A). The fact that all transgenic lines performed well suggests that the transgene configuration employed is less subject to position effects associated with the insertion sites in the mosquito genome [55,56].

IFA analysis corroborated the TCID_50_-IFA, demonstrating no tissue dissemination of virus for most transgenic lines, except BM2 and CM10, compared to wild-type HWE mosquitoes. Analysis of salivary CHIKV titers demonstrated the absence of virus in the pooled saliva of mosquitoes from transgenic lines BM8, BM16 and BM2, while a reduced titer of 7 × 10^1^ TCID_50_ /mL was present in the case of CM10 compared to the 1.5 × 10^6^ TCID_50_/mL viral titer obtained for HWE control mosquitoes (Figure 8). 

Because dissemination of transgenic mosquitoes will result in predominantly heterozygous offspring among the target population, a given antiviral transgene must be fully effective as a heterozygous allele. Direct PCR analyses of G_6_ transgenic females used for the infectivity assays were only partially homozygous, confirming the predominately heterozygous status of the hRz transgene among all tested female mosquitoes (Supplementary Table S7). We further confirmed the effectiveness of our ribozymes by challenging heterozygous G_9_ transgenic mosquitoes and demonstrating that only two of the lines yielded individual infected mosquitoes with viral titers three orders of magnitude lower than the wild-type control mosquitoes (Figure 7B). As a consequence, we can deduce that these hRz can be effective in a predominantly heterozygous population until transgene fixation is achieved. While our analyses do not address potential fitness costs associated with our transgene, at least one previous study has proposed that heterozygous transgenic mosquitoes are likely to exhibit reduced fitness costs relative to the native wild-type population [57]. These results suggest that appropriate expression of hRz antiviral transgenes might be used to eliminate the transmission potential of CHIKV in the natural vector. 

In this study we were able to demonstrate that hRzs can be effective as transgenic antiviral molecules and potentially could be useful in permitting replacement of competent vector populations with a CHIKV refractory vector population. However, since this report used the 181/25 laboratory strain of CHIKV, and the HWE laboratory strain of mosquitoes, additional analyses of these hRzs against natural CHIKV variants in native mosquito strains are needed. Additionally, for this technique to become practical it is important that the antiviral effector molecule remain functionally stable for multiple generations. If ultimately successful, this technique could potentially be used to reduce the spread of any arbovirus.

## Figures and Tables

**Table 1:** Template sequences used to amplify the t-RNA^val^ Pol III + hRz + PolyA_(60)_ expression cassettes for each hRz, and corresponding CHIKV target sites. The hRzs’ sequence is indicated in uppercase.

hRz	Template Sequence	Target Sequence (5’–3’)
hRz#9	cgaacccgggcactacaaaaaccaacaaCTATTTAGCTGATGAGGCCGAAAGGCCGAAACCGCCGTACgggcccgggcccaaaaa	gtacggcggtcctaaatag
hRz#10	cgaacccgggcactacaaaaaccaacaaAAGCGTCGCTGATGAGGCCGAAAGGCCGAAACTTCATGTGggggcccgggcccaaaaa	cacatgaagtccgacgctt
hRz#11	cgaacccgggcactacaaaaaccaacaaTTACGCGGCTGATGAGGCCGAAAGGCCGAAACCAGAGGGgggcccgggcccaaaaa	ccctctggtcccgcgtaa
hRz#12	cgaacccgggcactacaaaaaccaacaaAGCATGATCTGATGAGGCCGAAAGGCCGAAACTTGGTTTTggggcccgggcccaaaaa	aaaaccaagtcatcatgct
hRz#13	cgaacccgggcactacaaaaaccaacaaAATGGGTACTGATGAGGCCGAAAGGCCGAAACGCCGGTGAgggcccgggcccaaaaa	tcaccggcgtctacccatt
hRz#14	cgaacccgggcactacaaaaaccaacaaAGGCTGAACTGATGAGGCCGAAAGGCCGAAACATTGGCCCgggcccgggcccaaaaa	gggccaatgtcttcagcct
hRz#15	cgaacccgggcactacaaaaaccaacaaTCTTAGGGCTGATGAGGCCGAAAGGCCGAAACACATATACgggcccgggcccaaaaa	gtatatgtgtcccctaaga

## Supplementary Materials

Supplementary File 1
