# Supplementary material for: Antiviral Hammerhead Ribozymes Are Effective for Developing Transgenic Suppression of Chikungunya Virus in Aedes aegypti Mosquitoes
Source: Viruses. 2016 Jun 9;8(6):163. doi: 10.3390/v8060163 (PMC4926183; doi:10.3390/v8060163)
Supplement: Supplementary File 1 [file viruses-08-00163-s001.pdf]

Supplementary Materials: Antiviral Hammerhead Ribozymes Are Effective for Developing Transgenic Suppression of Chikungunya Virus in *Aedes aegypti* Mosquitoes

Priya Mishra, Colleen Furey, Velmurugan Balaraman and Malcolm J. Fraser, Jr.

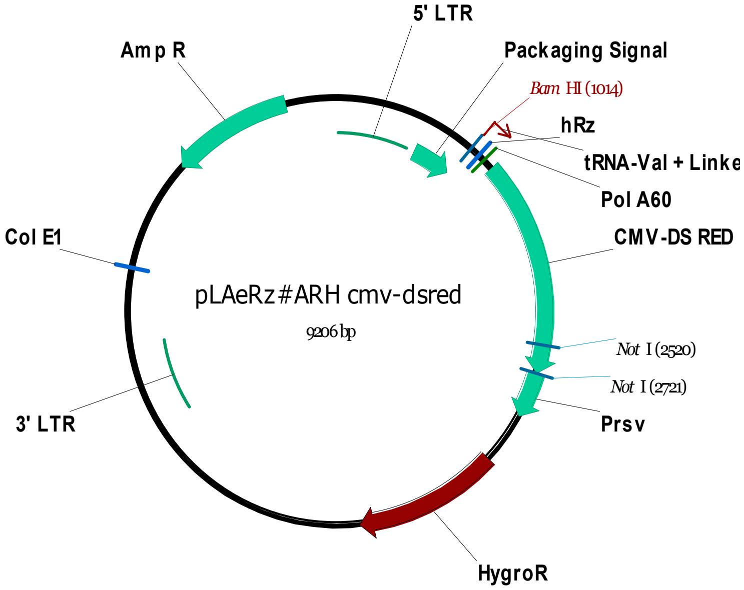

(**A**)

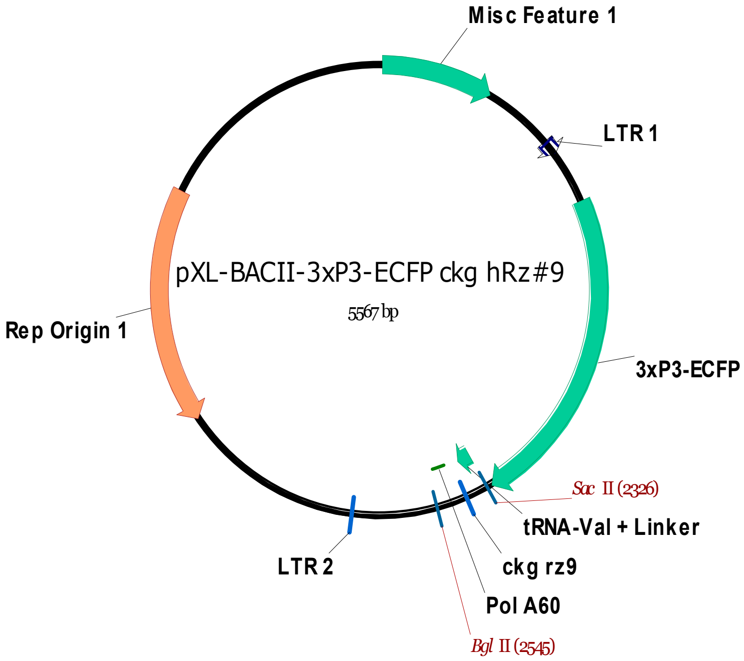

(**B**)

Figure S1. Plasmid maps: (**A**) pLAeRzARH plasmid used for the cloning of ribozymes. The plasmid was derived from pQCXIH by adding the RSV promoter to drive the independent expression of the hygromycin resistance gene. The transgene t-RNA^{Val} + hRzs + Pol A_{60} cloned using BamHI and NotI restriction sites.followed by the cloning of CMV-DsRed using PSpomI and NotI restriction sites. (**B**) *piggyBac* vector map containing hRz #9. Figure showing the location of tRNA^{val} hRz #9 + poly $A_{(60)}$ downstream of 3XP3-ECFP, cloned using SacII and BglII restriction sites.

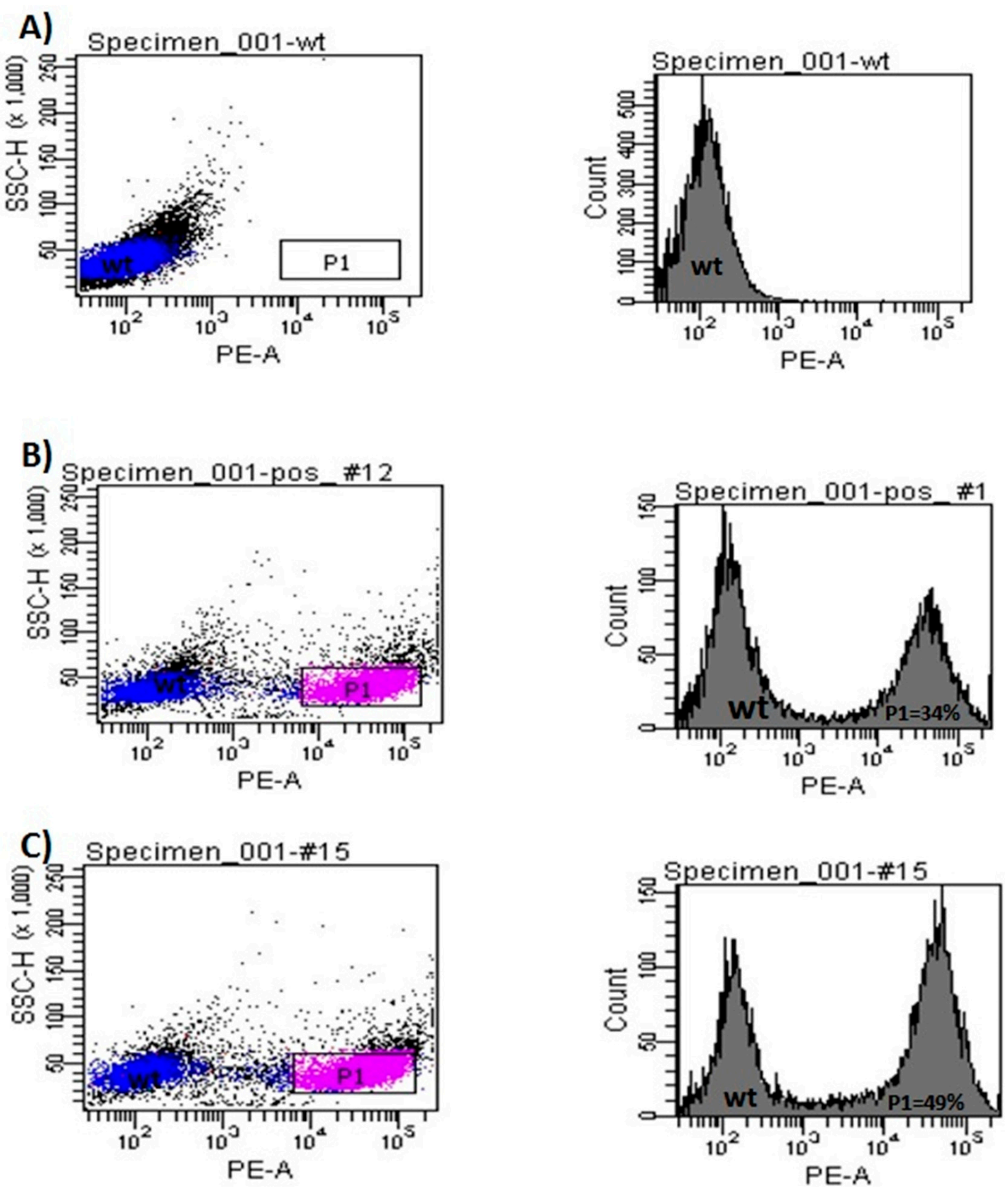

Figure S2. FACS sorting for establishing hRz transformed cell clones. (**A**) Untransformed Vero cells; (**B**) hRz #12 transfected and selected cells display 34% transformation (P1); (**C**) hRz #15 transfected and selected cells display 49% transformation (P1). P1 = percentage of transformed cells and wt = untransformed Vero cells.

Table S1. Reverse primer specific for each hRz, used in RT-PCR to detect the expression of each ribozyme in transformed cells.

**hRz**	**Reverse Primers**
#9	ataagaatgcggccgcgtttaacgtacggcggtttcggcctttcg
#10	ataagaatgcggccgcgtttaaccacatgaagtttcggcctttc
#11	ataagaatgcggccgcgtttaacccccctctggtttcggcctttc
#12	ataagaatgcggccgcgtttaacaaaacaagtttcggcctttcg
#13	ataagaatgcggccgcgtttaactcaccggcgtttcggcctttc
#14	ataagaatgcggccgcgtttaacgggccaatgtttcggcctttc
#15	ataagaatgcggccgcgtttaacgtatagtgtgtttcggcctttcg

Table S2. Primers used for direct PCR of mosquito transgenes.

Transgenic Line	Supercontig Number	Forward Primer	Reverse Primer
CM5	Supercont 1.150	tgggcgtttatgggtatagg	tatctacagggatcaccccg
BF3	Supercont 1.371	caacattccgatgatgcaaa	aacttttcccaaacggcttt
BM16	Supercont 1.607	cgcaaggttgtttggatttt	agaccaccaatcccctatcc
BM16	Supercont 1.373	tacggaattgcgttgaaaca	agacggggtacactttggaa
BF2	Supercont 1.1014	gtgacagcgagcaactctga	gaaaggattctcgacaggca
BM8	Supercont 1.94	ttgaagggaccatggaactc	aagtttgccgagagtcagct

Table S3. Establishment of transgenic mosquito lines from injected embryos.

Constructs	Total Injected	G_0 Adults	% Survival	Lines Obtained	Lines Established
Pxl-BacII-3xP3-ECFP-hRz#9	660	357	54%	9	7
Pxl-BacII-3xP3-ECFP-hRz#14	731	317	43%	3	2

Table S4. The total number of positive larvae obtained and the percentage transformation efficiency for each established transgenic line at G_1.

Transgenic Lines	Total Screened	Positives	% Transformation
CM5	548	6	1.1
CM10	345	5	1.5
BF5	1113	6	0.54
BF2	1026	16	1.6
BF4	939	14	1.5
BM2	946	14	1.5
BM8	618	7	1.1
BM16	1119	1	0.1
BF3	2495	3	0.1

Table S5. Splinkerette PCR analysis of integration sites of transgenes in the mosquito genome of each line: The supercontig each transgene insertion is shown, and, where possible, the chromosome number to which the supercontig has been mapped.

Transgenic Line	Chromosome Number	Supercontig Number
BF2 (5-BST)	unknown	1.1014
BF4 (5-BST)	unknown	1.121
BF5 (5-BAM)	unknown	1.131
BF5 (5-BFU)	unknown	1.17
CM5 (5-BAM)	unknown	1.5
CM10 (5-BFU)	unknown	1.64
CM10 (5-BST)	1p	1.4
BF3 (5-BAM)	unknown	1.172
BF3 (5-BGL)	unknown	1.371
BM2 (5-BGL)	unknown	1.3
BM16(5-BGL-BST)	unknown	1.607
BM16 (5-BAM)	unknown	1.373
BM8 (5-BAM)	3p	1.94

Table S6. Genes upstream and downstream of transgene insertions.

Transgenic Line	Integration Site in Known Gene	Nearest Upstream Gene	Nearest Downstream Gene
BF2 (5-BST)	no	kinesin heavy chain (36,317 bp)	Cannot find (greater than 70 kb)
BF4 (5-BST)	no	Conserved hypothetical protein (103,602 bp)	t-RNA ala (208,919 bp)
BF5 (5-BAM)	no	Conserved hypothetical protein (10,353 bp)	Conserved hypothetical protein (22,803 bp)
BF5 (5-BFU)	no	Fumaryl acetoacetate hydrolase (7763 bp)	Cannot find (greater than 80 kb)
CM5 (5-BAM)	no	Conserved hypothetical protein (1090 bp)	Hypothetical protein (4225 bp)
CM10 (5-BFU)	Yes, AAEL002681	Aldehyde oxidase (58,099 bp)	Vanin-like protein 1 precursor, putative (1595 bp)
CM10 (5-BST)	no	Hypothetical protein (172,193 bp)	Voltage-gated potassium channel (245,230 bp)
BF3 (5-BAM)	no	Conserved hypothetical protein (50,070 bp)	Conserved hypothetical protein (122,620 bp)
BF3 (5-BGL)	no	Cytochrome p450 (192,189 bp)	Cytochrome p450 (37,606 bp)
BM2 (5-BGL)	Yes (AAEL018225)	Conserved hypothetical protein (17,189 bp)	Conserved hypothetical protein (49 399 bp)
BM16 (5-BGL-BST)	Yes (AAEL011736)	Succinyl-coa synthetase beta chain (3928 bp)	Glutathione transferase (86,797 bp)
BM16 (5-BAM)	no	Rho GTPases activator (70,362 bp)	Cannot find (greater than 50 kb)
BM8 (5-BAM)	no	Ubiquinol-cytochrome c reductase iron-sulfur subunit (204,821 bp)	Cannot find (greater than 80 kb)

Table S7. Percentage heterozygosity for each transgenic line: PCR was performed on genomic DNA extracted from whole single mosquitoes at G_6.

Transgenic Line/ Supercontig Numbers	Total Number of Samples Used for PCR	Total Number of Samples Positive for PCR Product	Percentage Heterozygosity
BM16 (1.373)	21	10	48%
BM16 (1.607)	21	11	52%
BF2 (1.1014)	10	4	40%
BM8 (1.94)	18	18	100%
CM5 (1.5)	10	6	60%
BF3 (1.172)	18	7	39%

© 2016 by the authors. Submitted for possible open access publication under the terms and conditions of the Creative Commons Attribution (CC-BY) license (http://creativecommons.org/licenses/by/4.0/).
